# Correction: Activated K-ras and INK4a/Arf Deficiency Cooperate During the Development of Pancreatic Cancer by Activation of Notch and NF-κB Signaling Pathways

**DOI:** 10.1371/journal.pone.0101032

**Published:** 2014-06-17

**Authors:** The PLOS ONE Staff

There is an error in Figure 3A in the article; the Bcl2 lane in Figure 3A duplicates Notch 4 lane in Figure 1D. We are providing a revised Figure 3 with a corrected Bcl2 lane and the raw blots for each of the panels.

**Figure 3:**
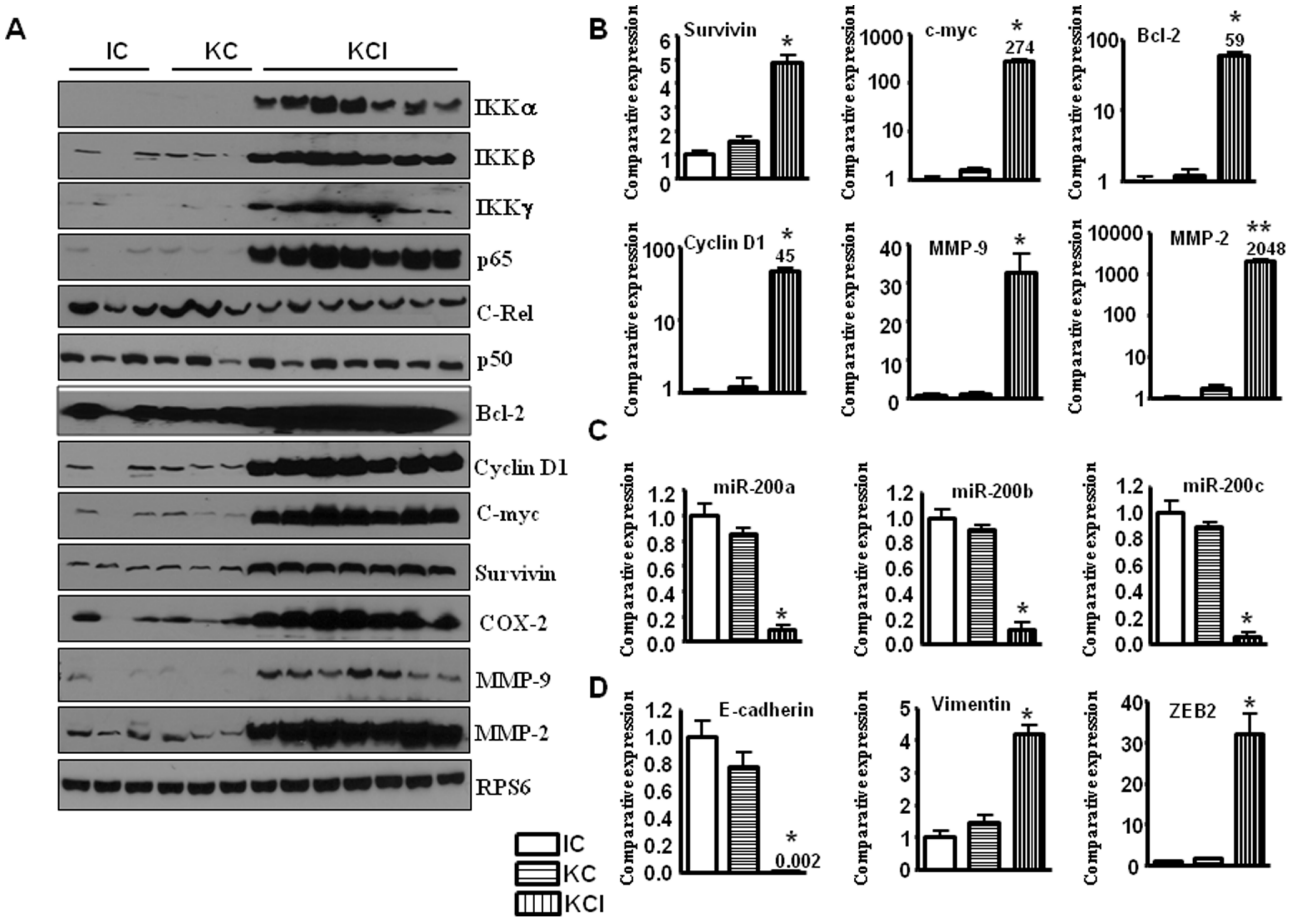
The expression of Notch target genesis increased in KCI mice. **A,** Western blot analysis showing the up-regulated expression of IKK, p65, and NF-*κ*B downstream genes in tumors derived from KCI mice. **B,** Real-time RT-PCR showing increased expression of NF-*κ*B downstream genes such as survivin, cyclin D1, Bcl-2,C-myc, MMP-2, and MMP-9 in the tumors derived from the KCI mice. **C,** The expression of miR-200 family was down-regulated in the tumors of the KCI mice as assessed by real-time RT-PCR. **D, **Real-time RT-PCR showing decreased expression of E-cadherin, and increased expression of vimentin, and a modest increase in the expression of ZEB1 whereas a 30-fold increased expression of ZEB2 in tumors derived from the KCI mice.

The authors regret this error. This mistake has no impact on the overall findings and conclusions reported in the article.

## Supporting Information

File S1 Raw blots for Figure 1D Notch-4
(TIF)

File S2 Raw blots for Figure 3ABcl-2
(TIF)
