# Supplementary figures and images for: Correction: Activated K-ras and INK4a/Arf Deficiency Cooperate During the Development of Pancreatic Cancer by Activation of Notch and NF-κB Signaling Pathways
Source: PLoS One. 2014 Jun 17;9(6):e101032. doi: 10.1371/journal.pone.0101032 (PMC4061083; doi:10.1371/journal.pone.0101032)

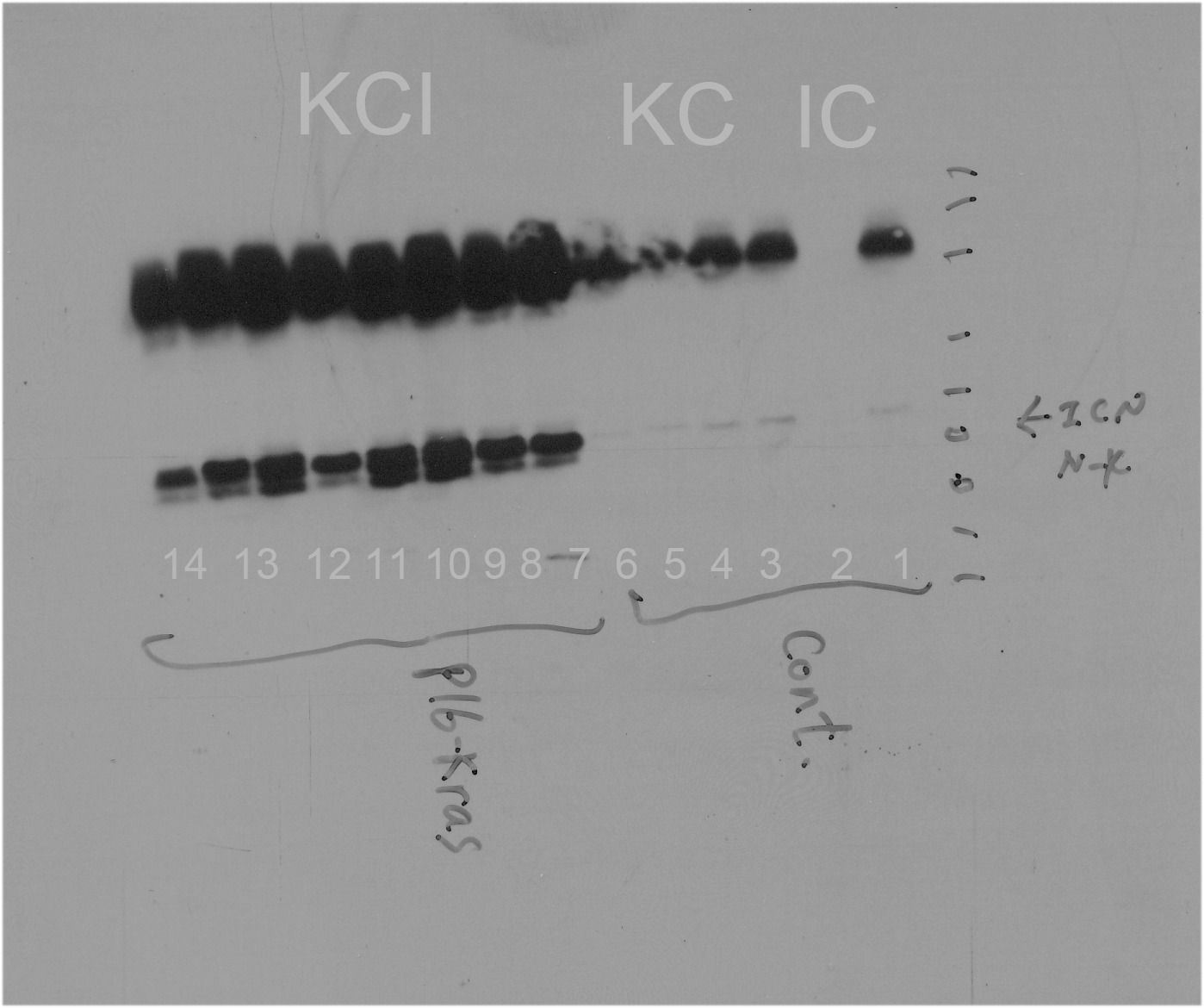

Supplement: File S1 — Raw blots for Figure 1D Notch-4 (TIF) [file pone.0101032.s001.tif]

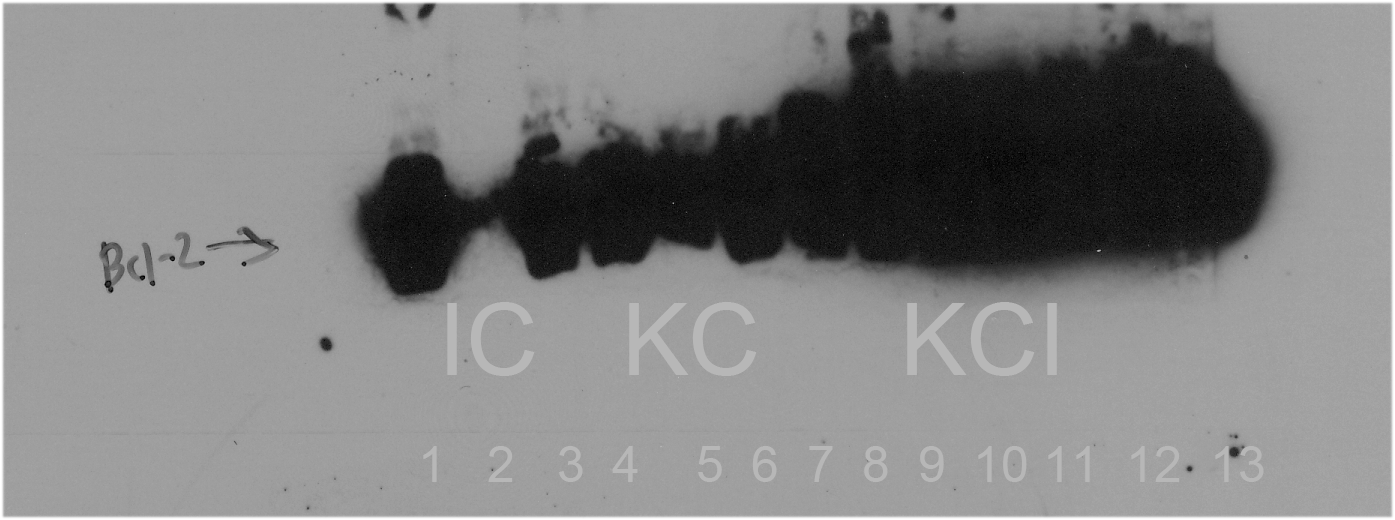

Supplement: File S2 — Raw blots for Figure 3ABcl-2 (TIF) [file pone.0101032.s002.tif]
